# Linking the Positivity Effect in Attention with Affective Outcomes: Age Group Differences and the Role of Arousal

**DOI:** 10.3389/fpsyg.2017.01877

**Published:** 2017-10-30

**Authors:** Cathleen Kappes, Berit Streubel, Kezia L. Droste, Kristian Folta-Schoofs

**Affiliations:** ^1^Department of Psychology, University of Hildesheim, Hildesheim, Germany; ^2^Department of Educational Psychology, University of Leipzig, Leipzig, Germany

**Keywords:** eye tracking, emotional reactivity, emotion regulation, well-being paradox

## Abstract

Despite its assumed importance for emotional well-being, studies investigating the positivity effect (PE) in older adults’ information processing rarely tested its relationship with immediate or general affective outcome measures like emotional reactivity or emotional well-being. Moreover, the arousal level of the to-be-processed emotional stimuli has rarely been taken into account as a moderator for the occurrence of the PE and its relationship with affective outcomes. Age group differences (young vs. old) in attention (i.e., fixation durations using eye tracking) and subjective emotional reactions (i.e., pleasantness ratings) were investigated in response to picture stimuli systematically varied in valence (positive vs. negative) and arousal (low vs. high). We examined whether there is a link between age group differences in fixation durations and affective outcomes (i.e., subjective emotional reactions as well as emotional well-being). Older compared to young adults fixated less on the most emotional part in negative but not in positive low-arousing pictures. This age difference did not occur under high arousal. While age group differences in fixation duration did not translate into age group differences in subjective emotional reactions, we found a positive relationship between fixation duration on negative low-arousing pictures and emotional well-being, i.e., negative affect. The present study replicated the well-known PE in attention and emotional reactivity. In line with the idea that the PE reflects top-down-driven processing of affective information, age group differences in fixation durations decreased under high arousal. The present findings are consistent with the idea that age-related changes in the processing of emotional information support older adults’ general emotional well-being.

## Introduction

Although older adults are thought to increasingly encounter losses in several life domains (Heckhausen et al., 1989; Baltes and Smith, 2003; Mustafić and Freund, 2012), they are still capable of maintaining relatively high levels of emotional well-being (Fiske et al., 2009; Charles and Carstensen, 2010; Wolitzky-Taylor et al., 2010; Scheibe and Carstensen, 2010; Carstensen et al., 2011). Socioemotional selectivity theory (SST; Carstensen et al., 1999, 2006; Carstensen, 2006), a prominent theory of emotional aging, posits that older adults are motivated to optimize their current affective experience and emotional well-being due to limits in future time perspective. More recently, the so-called “positivity effect” has been introduced as an important mechanism to explain the maintenance of emotional well-being among older adults. The term “positivity effect” (PE) refers to “a relative preference in older adults (compared to younger adults) for positive over negative material in cognitive processing” (Reed and Carstensen, 2012, p. 4). Within the framework of SST it is argued that the PE in older adults’ information processing reflects the recruitment of goal-directed, top-down driven processes in order to enhance, maintain or restore positive affective experience (Reed and Carstensen, 2012). Empirically, the PE has been particularly demonstrated in attention and memory using a wide range of experimental paradigms (e.g., eye tracking: Isaacowitz et al., 2006a,b; attention: Mather and Carstensen, 2003; and memory: Kennedy et al., 2004; Mikels et al., 2005; Mammarella et al., 2016; for a recent meta-analysis see Reed et al., 2014).

There is some evidence for the idea that the PE reflects cognitively controlled and thus resource demanding rather than automatic processes. Specifically, the PE has been shown to be enhanced in tasks supporting control-based processes (Mammarella et al., 2017). It was reduced or diminished among older adults with low cognitive functioning, when cognitive resources were experimentally depleted (Mather and Knight, 2005; Knight et al., 2007; Sasse et al., 2014), or during processing of high-arousing compared to low-arousing stimuli (Kensinger, 2008; Streubel and Kunzmann, 2011). Although the PE is thought to reflect the recruitment of goal-directed processes in order to optimize emotional well-being (e.g., Carstensen, 2006; Mather, 2012), the precise relationship between this age effect in cognitive processing and age-related changes in immediate or more general affective outcomes (i.e., subjective emotional reactions in response to the to-be-processed stimulus or emotional well-being) has rarely been studied (Isaacowitz and Blanchard-Fields, 2012).

In this study, we further investigated the PE in young and older adults by employing both measures of attention (i.e., fixation durations) and subjective emotional reactions (i.e., ratings of pleasantness and unpleasantness) in response to standardized positive and negative picture stimuli. By using these measures, we aimed to examine the precise relationship between age effects in attention, age effects in subjective emotional reactions, and age effects in general emotional well-being. We further examined a possible role of arousal for age group differences in attention and affective outcomes. To this end, we systematically varied the arousal level of the presented stimuli.

A common way to investigate the PE in attention is the registration of eye fixations (i.e., the duration of the eyes resting relatively stable) on (parts of) presented stimuli via eye-tracking. By using eye tracking, the PE has been demonstrated in a range of studies. In these studies, older compared to young adults fixated less on negative and more on positive faces (Isaacowitz et al., 2006a,b; Knight et al., 2007; Nikitin and Freund, 2011). Similarly, older adults fixated less on health messages with negative emotional content (Isaacowitz and Choi, 2012), and less on the most negatively judged part of unpleasant pictures (Noh et al., 2011). Furthermore, older adults demonstrating a gaze pattern predominantly to positive stimuli experienced less decrease in mood compared to older adults who did not exhibit such a preference (Isaacowitz et al., 2009b; Noh et al., 2011). Thus, several eye tracking studies provide evidence for the idea that age-related changes in the allocation of attention to emotional stimuli are indeed linked to changes in older adults’ affective experience (Isaacowitz, 2012). In line with the idea that the PE in attention reflects resource demanding processes, the relationship between gaze preferences and mood changes in older adults has been observed only for subjects with high cognitive functioning (i.e., executive control; Isaacowitz et al., 2009b).

The assumption that arousal moderates the PE in older adults’ information processing proceeds from the idea that distinct mechanisms are involved in the processing of low- and high-arousing emotional information. Whereas high-arousing stimuli primarily trigger automatic attentional capture to ensure prioritized processing (Dolan, 2002; Ferrari et al., 2008), enhanced processing of low-arousing stimuli involve top-down driven processes and thus require cognitive resources (Kensinger and Corkin, 2004; Mather and Knight, 2005; Knight et al., 2007). Given that the automatic processing of high-arousing information seems relatively preserved in old age (Mather and Knight, 2005), it may interfere with the goal-directed top-down driven information processing that underlies older adult’s PE. The idea that particularly low arousal enables the engagement of resource-demanding top-down processes in older adults is also in line with the Strength and Vulnerability Integration (SAVI) model (Charles, 2010). SAVI suggests that older adults have greater difficulties in modulating high levels of arousal once these are experienced. Accordingly, studies examining age differences in affective information processing as well as emotional reactivity demonstrated the PE exclusively or more pronounced in response to low-arousing vs. high-arousing stimuli (Kensinger, 2008; Streubel and Kunzmann, 2011; Dolcos et al., 2014; Kappes and Bermeitinger, 2016). For instance, Kensinger (2008) studied the PE in memory and showed that older adults remembered more positive than negative words when these were rated low in arousal, while young adults demonstrated the reverse pattern. However, no age difference in memory-retrieval was observed for high-arousing positive vs. negative words. Streubel and Kunzmann (2011) studied age differences in subjective emotional reactions and provide further evidence that the PE attenuates in the processing of high-arousing information. In their study, older adults rated positive pictures as more pleasant than young adults did, and this effect was noticeably more pronounced when pictures were low in arousal. Moreover, older adults rated negative low-arousing pictures as less unpleasant than young adults did, but there was no age difference in pleasantness ratings for high-arousing negative pictures.

In order to confirm and extend the existing findings on the PE, we identified three aims that might be of worth and of special interest:

(1) To replicate the well-described PE in attention we studied differences in younger and older adults’ eye fixations on positive and negative pictures chosen from the International Affective Picture System (IAPS; Lang et al., 2008). More precisely, we examined age group differences in fixation duration on defined emotional areas of interest (AOI; i.e., parts of the pictures that were judged as particularly emotionally relevant) relative to non-AOI parts of the pictures.
(2) To investigate the relationship between the PE in attention and age group differences in immediate as well as more general affective outcome measures, we additionally assessed subjective emotional reactions in terms of pleasantness ratings in response to the pictures as well as general emotional well-being. Most important, we examined the correspondence of age group differences in both affective outcome measures with age group differences in AOI-related fixations.
(3) To test arousal as a potential moderator of age group differences in fixation durations, affective outcomes and the precise relationship between these variables, we systematically varied the arousal of the given stimuli by presenting low- and high-arousing positive and negative pictures. We formulated the following predictions:

H1: *Age group differences in AOI-related fixation duration moderated by arousal.* For low-arousing pictures older adults were expected to fixate a larger percentage of time on emotionally relevant AOIs in positive compared to negative pictures. For young adults we predicted a reverse pattern, i.e., longer AOI-related fixation duration in negative than positive low-arousing pictures. For high-arousing pictures, we hypothesized these age group differences to diminish (at least partly or completely).
H2: *Age group differences in subjective emotional responses moderated by arousal.* We hypothesized age group differences in fixation duration to be reflected in subjective emotional responses. Hence, for low-arousing pictures, older adults were expected to react with less unpleasantness to negative pictures and with more pleasantness to positive pictures than young adults. Regarding high-arousing pictures, these age group differences were assumed to be less pronounced or even non-existent.
H3: *Age group differences in emotional well-being.* Given previous findings on emotional well-being (e.g., Charles and Carstensen, 2010; Carstensen et al., 2011), we expected older adults to report less general negative affect and more positive affect than young adults.
H4: *Relationship between AOI-related fixation duration and affective outcomes moderated by arousal.* Given the idea that age-related differences in information processing reflect older adults’ attempts to optimize their current affective experience, we expected that age group differences in AOI-related fixation duration are associated with age group differences in subjective emotional responses as well as emotional well-being. Assuming that arousal moderates the occurrence of age group differences in information processing, we expected the aforementioned relationships between fixation duration and affective outcomes to occur only for low-arousing pictures but not for high-arousing pictures.

## Materials and Methods

### Participants

Twenty-one young adults (*N* = 19 women, 19–28 years, *M*_age_ = 21.29 ± 2.31) and nineteen older adults (*N* = 13 women, 59–77 years, *M*_age_ = 69.78 ± 5.98) participated in this study. We expected medium to large statistical effects for interactions between age group, valence and arousal based on previous findings regarding fixation duration (Isaacowitz et al., 2006a; Knight et al., 2007) and emotional reactivity (Streubel and Kunzmann, 2011). Unfortunately, G^∗^Power (Erdfelder et al., 1996) is not suited to calculate a priori power analyses for designs with two within-subject factors and one between-subject factor. A power-analysis for a 2 × 2 design [e.g., age group (between) × valence (within)] indicated that we required *N* = 16 (large effect) to *N* = 34 (medium sized effect) participants in total to achieve 80% power when employing the traditional 0.05 criterion of statistical significance. For the correlational analyses G^∗^Power indicated that we would need *N* = 27 to *N* = 81 participants to achieve 80% power to detect large to medium sized effects with 0.05 as criterion for statistical significance. Moreover, assuming large effect sizes for the mediational analysis, Fritz and MacKinnon (2007) suggest *N* = 34 to have 80% power for detecting a significant bias-corrected bootstrapped coefficient. The sample size accords with a priori power analyses for the expected large effect sizes. Still, we aimed at a larger sample to increase chances to detect only medium-sized effects. Unfortunately, the study had to be conducted in a quite narrow time slot and recruitment of (especially older) participants was also difficult. Participants were recruited at the university campus (older adults: guest auditor program for elder persons), adult education centers, via local newspaper advertisements, lists of former participants and student lists. All participants were highly (German *Abitur)* or intermediately (*Mittlere Reife*, equivalent to high school level) educated (young adults: 100% *Abitur*; older adults: 39% *Abitur*, 44% *Mittlere Reife*). While young adults received course credit for participation, older adults received no compensation. Young and older adults reported to be healthy, and to have normal or corrected-to-normal vision. Older adults judged their vision and hearing to be slightly worse than young adults (vision: *M*_young_ = 4.6, *M*_old_ = 3.7, *t*(38) = 2.69, *p* < 0.05; hearing: *M*_young_ = 4.2, *M*_old_ = 3.5, *t*(38) = 3.84, *p* < 0.05; all items ranging from “1” [*very bad*] to “5” [*very good*]). In addition, there were no significant age group differences with respect to self-reported mobility (*M*_young_ = 4.4, *M*_old_ = 4.0) or fitness (*M*_young_ = 3.8, *M*_old_ = 3.3). This study was carried out in accordance with the recommendations of the ethics committee of the German Psychological Society. All subjects gave written informed consent in accordance with the Declaration of Helsinki. The protocol was approved by the local ethical committee of the University of Hildesheim.

### Stimuli and Apparatus

Stimuli were 113 colored pictures selected from the International Affective Picture System (IAPS, Lang et al., 2008). In previous studies, non- or low-arousing stimuli ranged from a very low to a medium level of arousal (Kensinger, 2008; Streubel and Kunzmann, 2011). For example, Kensinger (2008) classified stimuli as non-arousing when rated between 1 and 4.9 on a scale ranging from 1 (*low*) to 9 (*high-arousing*). To delineate the influence of arousal and to avoid the mixture of non-arousing (i.e., neutral) and low-/medium-arousing stimuli, we narrowed the arousal range for low-arousing stimuli from 2.0 to 4.5 in comparison to high-arousing (>4.5) positive and negative stimuli. Based on ratings assessed in younger and older German adults in prior studies (Grühn and Scheibe, 2008; Streubel and Kunzmann, 2011), pictures were selected and grouped into five affective stimulus-categories: *neutral* (e.g., domestic utensils; *M*_arousal_ = 1.0 to 2.0; *M*_valence_ = 4.0 to 6.0), *low-arousing positive* (e.g., family trips, nature; *M*_arousal_ > 2.0 to 4.5; *M*_valence_ > 6.0), *low-arousing negative* (e.g., insects, pollution; *M*_arousal_ > 2.0 to 4.5; *M*_valence_ < 4.0), *high-arousing positive* (e.g., erotica, skydiving; *M*_arousal_ > 4.5; *M*_valence_ > 6.0), and *high-arousing negative* (e.g., illness, death; *M*_arousal_ > 4.5; *M*_valence_ < 4.0). Each affective category contained 8 pictures. The neutral category contained a total of 32 pictures. All pictures were comparable in luminance and matched in arousal and valence levels for both age groups (see **Table 1** for descriptive statistics; see Appendix B for list of IAPS numbers).

**Table 1:** Descriptive statistics: normative ratings of arousal and valence by stimulus category and age group.

		Young adults					Older adults			
		*N*	Minimum	Maximum	*M*	*SD*	Minimum	Maximum	*M*	*SD*
Neutral	Arousal	32	2.16	2.93	2.56	0.22	2.15	2.91	2.61	0.20
	Valence	32	4.56	5.96	5.40	0.36	4.85	6.00	5.61	0.28
Low-arousing positive	Arousal	8	3.73	4.15	3.91	0.17	3.82	4.10	3.95	0.11
	Valence	8	6.40	7.19	6.61	0.26	6.15	8.71	7.42	0.92
High-arousing positive	Arousal	8	4.78	5.41	5.07	0.20	4.65	5.53	5.09	0.35
	Valence	8	6.13	7.10	6.68	0.37	6.15	8.08	7.08	0.64
Low-arousing negative	Arousal	8	3.74	4.44	4.05	0.27	3.73	4.44	4.11	0.24
	Valence	8	2.43	3.92	3.38	0.62	2.72	3.59	3.30	0.27
High-arousing negative	Arousal	8	4.93	5.19	5.08	0.10	4.81	5.30	5.08	0.18
	Valence	8	3.07	3.89	3.41	0.27	2.93	3.92	3.49	0.37

We identified emotional AOIs of the pictures based on a separate online rating study, conducted on 134 German students who did not participate in the current study. Therefore, all 113 pictures were divided into two blocks. Pictures of block A (*N* = 57) were rated by 69 participants (52 women, *M*_age_ = 22.25 ± 4.41 years); pictures of block B (*N* = 56) were rated by 65 participants (46 women, *M*_age_ = 22.42 ± 4.53 years). A grid of 48 squares was superimposed on each picture (see Appendix A). Participants were asked to identify on each picture an area consisting of four contiguous squares that they judge as highly emotionally meaningful (i.e., as the most negative parts of negative pictures and the most positive part of positive pictures). Each picture allowed a total of 35 possible combinations of four contiguous squares. AOIs were identified based on the most frequently chosen combination of four coherent squares for each picture. As female participants were overrepresented in this sample (73% female), we weighted to adjust for the unequal gender distribution. Participants rated the valence for each of their selected AOIs on a scale ranging from “1” (*extremely unpleasant*) to “9” (*extremely pleasant;* see Appendix B).

Stimuli were displayed on a 24 inch TFT-monitor (1920 pixel × 1200 pixel, 60 Hz, 12 ms S/W). The background of the monitor was set to gray. Eye movements were recorded for the left monocular eye using an infrared high-speed eye tracking system (iViewX^TM^ Hi-Speed 1250 Hz System; SensoMotoric Instruments, Teltow, Germany) with high temporal (1250 Hz) and spatial resolution (0.25–0.5° visual arc). Recording of eye movements was controlled by a PC (Intel Core 2 Duo Processor, 2.66 GHz; system software: Windows XP, Version 2002), and by the recording software iViewX (Version 2.0.23b; SensoMotoric Instruments, Teltow, Germany). A network connection allowed for the exchange of stimulus-trigger between the stimulus computer and the recording system. In the beginning of the experiment, a calibration of the eye tracking system was conducted using a nine-point calibration matrix.

### Procedure

Prior to the experiment, participants signed informed consent. To assess general emotional well-being, participants were asked to complete a German translation of the Positive and Negative Affect Schedule (PANAS; Krohne et al., 1996) at a laptop using E-prime 1.1 (Psychology Software Tools, Inc., Pittsburgh, PA, United States). To this end, participants indicated on a 5-point Likert-scale (ranging from 1 = *never* to 5 = *very often*) how frequently they had experienced each of the listed positive and negative emotions during the last year. The positive affect scale consisted of 10 positive emotions (e.g., interested, excited) and showed high internal consistency (Cronbach’s *α* = 0.81). The same held true for the 10 items of the negative affect scale (e.g., distressed, irritated; Cronbach’s *α* = 0.78).

Measurement of eye-movements and recording of immediate emotional responses took place in a sound-attenuated and dimly illuminated chamber (2 m × 4 m × 2.5 m). The diffuse illumination of the chamber was adopted to the luminance of the screen (50 cd/m^2^). Participants sat in front of a monitor on a height-adjustable chair and used a height-adjustable chin-rest and a forehead support to ensure stable fixation on the screen. Viewing distance was 100 cm. Participants were informed that they would be viewing a slide show of photographs. They were instructed to view the images as if at home watching television or viewing photographs. Further, they were informed that they would be asked to report how they felt when viewing the picture. Pictures were presented centrally for a duration of 4 s and in a randomized order. To assess subjective emotional reactions, participants were asked, consecutively after each picture, to rate their feelings of pleasantness and arousal while viewing the picture via a digitized version of the Self-Assessment Manikin (SAM; Bradley and Lang, 1994). SAM depicts graphic figures representing different levels of experience of pleasantness and arousal, respectively. The anchors of both 9-point rating scales were labeled with “1” (*unpleasant* and *calm*, respectively) and “9” (*pleasant* and *exiting*, respectively). Participants responded by pressing defined keys on a standard numerical keyboard with the middle or index finger of their dominant hand. Before each picture, a white fixation cross (20 pixels) was presented centrally for a duration of 1 s and participants were instructed to relax and to clear their mind of any thoughts, emotions, or memories.

### Eye Tracking Data Analysis

We analyzed eye tracking data using BeGaze 3.4.27 (SensoMotoric Instruments, Teltow, Germany). This analysis software allowed for an automatic detection and exclusion of blinks, and for the extraction of saccade-like events (SLE) and fixations using a velocity-based, high-speed event detection algorithm. Saccades were defined as SLE, when peak velocity exceeded a threshold of 80°/s in a time window comprising 20–80% of the duration of a SLE. Additionally, a saccade duration of at least 25 ms, followed by a fixation duration of at least 100 ms were required (Rayner, 1998; Manor and Gordon, 2003; Folta-Schoofs et al., 2015). Accordingly, fixations were defined as the time between the end of one SLE and the beginning of the next one (Galley et al., 2015).

## Results

### Arousal as a Moderator of Age Group Differences in AOI-Related Fixation Duration

In our sample of pictures, the neutral picture category was only included to exclude possible effects of habituation to emotional stimuli. Given that the hypotheses of this study did not refer to neutral pictures, data from this picture category had been excluded from all statistical analyses.

In a first step, we aggregated fixation duration across pictures of each picture category (low- and high-arousing negative vs. positive pictures). Second, to test age group differences in total fixation duration on the whole picture and to verify whether participants followed the instruction to look at the pictures, we conducted a mixed ANOVA with valence (negative vs. positive) and arousal (low vs. high) as within-subjects factors, age group (young vs. old) as between-subjects factor and total fixation duration on the whole picture as the dependent variable. There was a significant age group difference in total fixation duration, *F*(1,38) = 12.49, *p* = 0.001, $\eta_{p}^{2}$ = 0.25. Generally, older adults fixated shorter than young adults on the whole picture (*M*_old_ = 3,046 ms, *M*_young_ = 3,433 ms). Given that pictures were presented for a duration of 4 s, the resulting average fixation durations were considered as reasonably long to indicate that participants followed the instruction. No other main effects or interactions with age group were observed, but there was a significant interaction between valence and arousal, *F*(1,38) = 7.92, *p* = 0.008, $\eta_{p}^{2}$ = 0.17. Independent from their age, participants fixated high-arousing negative pictures (*M* = 3,295 ms) longer than low-arousing negative pictures (*M* = 3,227 ms). Contrary, they fixated low-arousing positive pictures (*M* = 3,257 ms) longer than high-arousing positive pictures (*M* = 3,180 ms). In order to control for age group differences in total fixation duration, we calculated AOI-related fixation duration as percentage of time spent on the emotionally relevant AOIs relative to time spent on the rest (non-AOI parts) of the corresponding picture.

**FIGURE 1:**
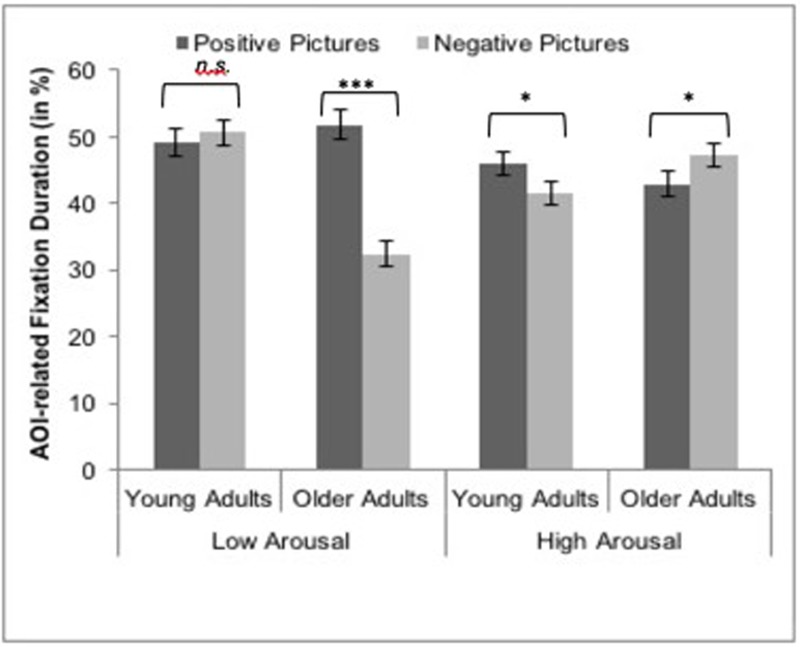
Means (± standard errors) of young (*N* = 21) and older (*N* = 19) adults’ AOI-related fixation duration (i.e., percentage of fixation duration on AOIs relative to the rest of the picture) for low- and high-arousing positive and negative pictures. ^∗^*p* < 0.05, ^∗∗∗^*p* < 0.001.

We examined age group differences in AOI-related fixation duration with a mixed ANOVA comprising valence (negative vs. positive) and arousal (low vs. high) as within-subjects factors and age group (young vs. old) as between-subjects factor. Significant main effects of valence and arousal as well as their interactions with age group were qualified by a significant 3-way interaction, *F*(1,38) = 62.16, *p* < 0.001, $\eta_{p}^{2}$ = 0.62 (see **Figure 1**). In order to dissect this interaction, we separately conducted mixed ANOVAs for low- and high-arousing pictures, followed up by paired *t*-tests for young and older adults, respectively. Both the ANOVAs for low- as well as high-arousing pictures revealed a significant valence-by-age group interaction, *F*_low_(1,38) = 60.85, *p* < 0.001, $\eta_{p}^{2}$ = 0.62; *F*_high_(1,38) = 9.57, *p* < 0.001, $\eta_{p}^{2}$ = 0.20. Consistent with our predictions for low-arousing pictures, *post hoc* comparisons revealed that older adults fixated emotionally relevant AOIs (in comparison to the rest of the picture) longer in positive than in negative pictures [*t*(18) = -10.32, *p* < 0.001; *M*_pos_ = 51.9%, *M*_neg_ = 32.4%]. In contrast, there was no significant difference in AOI-related fixation duration for low-arousing positive and negative pictures in young adults, [*t*(20) = 0.76, *p* = 0.45 (*M*_pos_ = 49.2%, *M*_neg_ = 50.6%]. As expected, this preference in AOI-related fixations for positive (vs. negative) pictures in older adults did not occur in response to high-arousing pictures. That is, older adults fixated emotionally relevant parts longer in negative than positive high-arousing pictures [*M*_pos_ = 42.9%, *M*_neg_ = 47.2%; *t*(18) = 2.23, *p* = 0.042], while young adults fixated proportionately longer on these AOIs in positive than negative high-arousing pictures [*M*_pos_ = 46.1%, *M*_neg_ = 41.5%; *t*(20) = -2.17, *p* = 0.038].

### Age Group Differences in Affective Outcomes

#### Arousal as a Moderator of Age Group Differences in Subjective Emotional Responses

**FIGURE 2:**
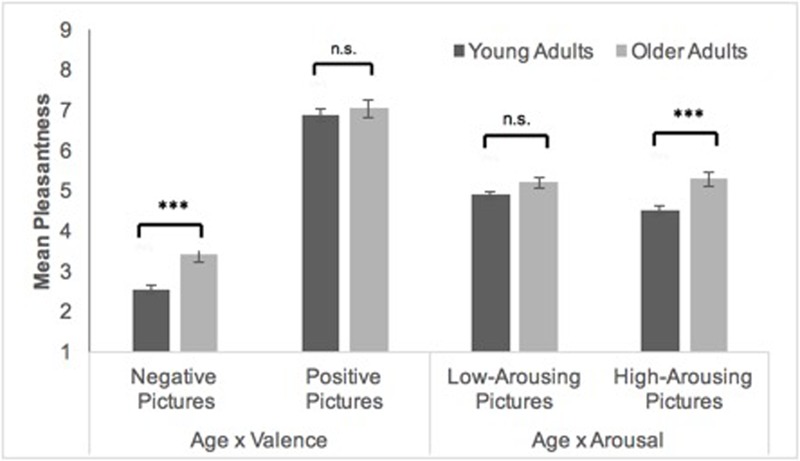
Means (± standard errors) of young (*N* = 21) and older (*N* = 19) adults’ pleasantness ratings for negative and positive pictures and for low- and high-arousing pictures. ^∗∗∗^*p* < 0.001.

A mixed ANOVA with valence (positive vs. negative) and arousal (low vs. high) as within-subjects factors, age group (young vs. old) as between-subjects factor, and pleasantness ratings as the dependent variable revealed significant main effects of age group, *F*(1,38) = 10.11, *p* = 0.003, $\eta_{p}^{2}$ = 0.21, and valence, *F*(1,38) = 529.27, *p* < 0.001, $\eta_{p}^{2}$ = 0.93, qualified by significant interactions between age group and valence, *F*(1,38) = 4.45, *p* = 0.041, $\eta_{p}^{2}$ = 0.11, as well as age group and arousal, *F*(1,38) = 5.36, *p* = 0.026, $\eta_{p}^{2}$ = 0.12. Following-up on these interactions revealed that older compared to young adults experienced negative pictures as more pleasant [*M*_young_ = 2.53; *M*_old_ = 3.43, *t*(38) = -3.92, *p* = 0.001], whereas the age groups did not differ in their affective experience in response to positive pictures [*M*_young_ = 6.88; *M*_old_ = 7.04, *t*(38) = -0.67, *p* = 0.51; see **Figure 2**]. Moreover, both age groups experienced low-arousing pictures as similarly pleasant [*M*_young_ = 4.89; *M*_old_ = 5.20, *t*(38) = -1.70, *p* = 0.098]. In contrast, older compared to young adults rated high-arousing pictures as more pleasant [*M*_young_ = 4.51; *M*_old_ = 5.28, *t*(38) = -3.61, *p* = 0.001]. This was due to younger adults’ lower rating of high-arousing compared to low-arousing pictures, *t*(20) = 2.61, *p* = 0.017.

#### Age Group Differences in Emotional Well-Being

*T*-tests for positive and negative affect revealed the expected age group difference for negative affect. That is, during the last year older adults experienced significantly less negative affect than young adults, *t*(37) = 2.23, *p* = 0.032 (*M*_old_ = 2.27, *M*_young_ = 2.58; *d*_Cohen_ = -0.72). The age groups did not significantly differ regarding positive affect, *t*(37) = -1.31, *p* = 0.20 (*M*_young_ = 3.82, *M*_old_ = 4.02).

### Relationship between AOI-Related Fixation Duration and Affective Outcomes

To test the correspondence of age group differences in affective outcomes with age group differences in AOI-related fixation duration, we first calculated Pearson correlations separately for each picture category between AOI-related fixation duration and subjective emotional responses as well as between AOI-related fixation duration and emotional well-being (see **Table 2**). Regarding immediate emotional responses, there were no significant relationships with AOI-related fixation duration, *p*s > 0.28. Because of an apparent lack of a significant relationship between fixation duration and subjective emotional responses, we did not conduct mediation analyses to test the role of age group differences in fixation duration for age group differences in subjective emotional responses.

**Table 2:** Correlations between AOI-related fixation duration, pleasantness ratings and emotional well-being by picture category.

	AOI-related fixation duration			
	Positive pictures		Negative pictures	
	Low-	High-	Low-	High-
	arousing	arousing	arousing	arousing
Pleasantness ratings	0.03	0.11	-0.15	0.18
PA	-0.07	-0.13	-0.12	0.23
NA	-0.04	0.06	0.37^∗^	-0.14

*PA, general positive affect; NA, general negative affect. ^∗^*p* < 0.05.*

An analysis of the relationship between fixation duration and emotional well-being revealed no significant relationship between fixation duration and positive affect. However, as expected, there was a significant correlation between the duration of AOI-related fixations on low-arousing negative pictures and negative affect, *r*(38) = 0.37, *p* = 0.022, which was not observed for high-arousing negative pictures [*r*(38) = -0.14, *p* = 0.38; *z* = 2.24, *p* < 0.05; Lee and Preacher, 2013]. That is, participants who fixated less long on the emotionally relevant AOIs of low-arousing negative pictures reported less negative affect during the last year. To test whether age group differences in negative affect were associated with age group differences in AOI-related fixation duration for low-arousing negative pictures, we conducted a mediation analysis employing PROCESS (Hayes, 2013; model 4) with age group as the predictor, AOI-related fixation duration as the mediator and negative affect as the criterion. As expected, the effect of age group on negative affect (*B* = -0.31, *p* < 0.05) decreased after inclusion of fixation duration and became non-significant (*B* = -0.15, *p* = 0.46); however, the indirect effect of age group via fixation duration was not significant (*B* = -0.16, 95% bootstrapped confidence interval = -0.45, 0.04; Sobel test: 1.07, *p* = 0.28).

Finally, we tested in an exploratory manner, whether both age groups differed significantly in the strength of the relationship between AOI-related fixation duration and affective outcome measures using software provided by Preacher (2002). Concerning general negative affect, young adults did not show any significant correlation with fixation duration. In contrast, older adults showed more pronounced positive correlations between negative affect and fixation duration at emotionally relevant AOIs on negative low-arousing pictures, *r*(17) = 0.49, *p* = 0.038. However, this age group difference did not reach significance [*r*_young_(19) = 0.23, *p* = 0.308; *z* = -1.60, *p* = 0.11]. All other correlation coefficients in both age groups were non-significant (*p*s > 0.21).

## Discussion

The present study examined age group differences in the processing of emotional stimuli. Therefore, we employed high-speed eye tracking while young and older adults viewed positive and negative IAPS pictures that differed in their arousal. We investigated how the PE in attention, as indexed by age group differences in fixation duration during picture viewing, is related to immediate as well as more general affective outcome measures, that is, experience of pleasantness in response to emotional pictures and emotional well-being.

### Age Group Differences in AOI-Related Fixation Duration Moderated by Arousal

In the present study, we replicated the well-known PE in fixation patterns during emotional picture viewing (Isaacowitz et al., 2006a,b). Additionally, the present results support and extend previous work on the PE in affective information processing (i.e., memory and emotional reactions, Kensinger, 2008; Streubel and Kunzmann, 2011; Mammarella et al., 2016), because they suggest that the PE in attention is reduced under conditions of high arousal. Consistent with our predictions, older adults fixated less on the most emotionally relevant parts in negative compared to positive low-arousing pictures, whereas young adults did not show such a difference under conditions of low arousal. In contrast, in response to high-arousing pictures, older adults fixated longer on the most emotionally relevant parts in negative than positive pictures, whereas young adults demonstrated the reversed pattern. Our findings emphasize the importance of arousal as a moderator for age differences in the processing of affective information.

Moreover, our findings contribute to an ongoing debate whether the PE can be considered to result from cognitively controlled processes (Mather and Knight, 2005; Reed and Carstensen, 2012), cognitive decline (Labouvie-Vief et al., 2010) or age-related impairments in amygdala function leading to reduced neural and affective responses to negative, but not to positive stimuli (Cacioppo et al., 2011). If the PE predominantly depends on cognitive decline, the PE is expected to become visible particularly when being confronted with high-arousing stimuli, because processing of negative stimuli taxes cognitive resources more (as compared to positive information) and should be particularly diminished under conditions of high arousal (see also Charles, 2010). Similarly, if the PE predominantly depends on impaired amygdala functions, it is also expected to be larger for high-arousing stimuli, because processing of these stimuli predominantly relies on information processing in limbic networks. Our findings better fit in with the idea that the PE is the result of top-down driven cognitive processes (Mather and Knight, 2005; Knight et al., 2007; Sasse et al., 2014; Kalenzaga et al., 2016; Mammarella et al., 2016, 2017). Implementation of these processes may be impeded in situations involving high arousal most likely resulting in the diminishment of the PE.

### Age Group Differences in Affective Outcomes

#### Age Group Differences in Emotional Well-Being

Consistent with our predictions and previous findings (Charles and Carstensen, 2010; Carstensen et al., 2011; see review by Scheibe and Carstensen, 2010), older compared to young adults reported less general negative affect and a comparable extent of positive affect during the last year, supporting the assumption that emotional well-being remains relatively stable or even improves in older adulthood.

#### Arousal as a Moderator of Age Group Differences in Subjective Emotional Responses

Age group differences in immediate emotional responses to emotional pictures concur with these findings in that older compared to young adults experienced negative pictures as less unpleasant, whereas no age group difference for positive pictures occurred. Contrary to our hypothesis and findings from Streubel and Kunzmann (2011), this interaction between age group and valence was independent of the pictures’ arousal level. One explanation for this inconsistency between both studies concerns differences in the mean arousal level of employed stimuli in both studies. Streubel and Kunzmann (2011) used high-arousing negative stimuli with higher mean arousal and low-arousing positive stimuli with lower mean arousal compared to the present study. The smaller difference between the mean arousal of the low- and high-arousing picture categories in the present study might have reduced the impact of arousal on age differences in emotional responses obtained by Streubel and Kunzmann (2011).

Another explanation concerns differences in the duration of stimulus presentation. Streubel and Kunzmann (2011) presented pictures longer (i.e., 6 s) than in the present study (4 s). As will be discussed in more detail in the section below, it might be possible, that it took a certain amount of time for cognitive regulation processes to “translate” into immediate benefits in emotional experiences that was not sufficiently allowed in the present study.

### Relationship between Fixation Duration and Affective Outcome Measures Moderated by Arousal

There were no meaningful relationships between fixation duration on the most positive or most negative parts of the positive or negative pictures and subjective emotional reactions (i.e., pleasantness ratings) in response to the pictures. This lack of meaningful relationships between attention allocation during picture processing and immediate emotional reactivity suggests that differences in the processing of emotional information do not relate to differences in immediate emotional reactions – at least not always or not as fast. Thus, age group differences in fixation duration cannot account for age group differences in immediate emotional reactivity in the present study. It might be possible that 4 s of stimulus presentation are not sufficient for top-down information processing mechanisms to translate into emotional outcomes. Isaacowitz et al. (2009a) demonstrated that gaze preferences for neutral over angry faces in older adults only emerged after a time-interval of 3 s. However, they did not test the relationship between gaze patterns and immediate emotional outcomes. In the same vein, Allard and Kensinger (2014) suggested that older adults activated emotion regulatory processes (particularly reappraisal processes) only during the emotional peak of a film clip. Accordingly, it might take several seconds until emotion regulatory processes, as they might be reflected in fixation patterns, become mirrored in emotional responses (see also Scott et al., 2017, for an even longer time course for age differences to be observed).

The present findings regarding the relationship between fixation patterns and general affective outcome measures, i.e., emotional well-being, lend support for this interpretation. Consistent with our prediction, fixation durations on the most negative parts of negative pictures were positively associated with negative affect. As expected, the effect of age group in negative affect markedly decreased on a descriptive level when including fixation duration as predictor suggesting that age group differences in emotional well-being partially rely on attentional processes. However, the indirect effect of age group via fixation duration on negative affect turned out to be not significant, which may be due to the small sample size. Future studies are needed to strengthen this interpretation.

More importantly, we found the advantageous relationship between attentional avoidance of the most unpleasant parts of negative pictures and emotional well-being (i.e., less negative affect) only with respect to low-arousing material. Additionally, we tested whether the relationship between fixation duration and affective outcome measures differed between age groups and found that the positive relationship between fixation duration on negative low-arousing pictures and general negative affect only held within the older age group. These findings underline the assumption that low-arousing stimuli allow the implementation of top-down driven processes of a pro-hedonic orientation, while high-arousing stimuli evoke automatic processing and limit the implementation of top-down-driven processes presumably underlying the PE. Moreover, in line with a prior eye-tracking study our findings lend support for the idea that not only the PE *per se*, but also the “translation” of the PE in information processing into emotional outcomes (i.e., less negative or more positive affect) rely on resource demanding processes. In this study, only older adults with high cognitive functioning have been shown to benefit from a relative preference in fixation patterns for positive over negative pictures, in that they sustained positive mood during the experiment. In contrast, older adults with reduced cognitive functioning showed significant declines in mood even though they showed a similarly positive preference in fixation patterns (Isaacowitz et al., 2009b; Noh et al., 2011).

Furthermore, our findings suggest that the implementation of attentional avoidance is particularly beneficial in the older age group, given that fixation duration was unrelated to emotional well-being in younger adults. Alternative regulatory processes such as reappraisal might be more relevant or effective in the younger age group (Urry and Gross, 2010).

### Caveats and Outlook

As discussed, the present findings suggest that age-related changes in the top-down driven processing of emotional information might support older adults’ emotional well-being. Future work is needed to better understand if and under which conditions age-related changes in affective information processing actually benefit immediate emotional experience. As discussed, the time between the assessment of attention allocation and emotional reactions might play an important role for regulatory processes to translate into emotional outcomes. It will be interesting to systematically vary the duration of stimulus presentation or employ online measures of emotional experience to assess the time course it needs for cognitive processes to be reflected in emotional responses. Additionally, the direction of the relationship between regulatory processes and emotional well-being cannot be determined with the present design. The inclusion of longitudinal measurements may help to understand the direction of the influence of regulatory processes on emotional well-being and vice versa.

A second limitation pointed out by Freund and Isaacowitz (2014) refers to the problem of employing extreme age group comparisons as an approximation to investigate age-related changes as they may lead to overestimation of age-related effects and are blind to changes in middle adulthood. Moreover, age is merely used as a proxy variable for underlying psychological processes (i.e., changes in the motivation to optimize one’s current affective experience and emotional well-being) that cause the observed age group differences (e.g., in fixation patterns or emotional reactivity). Thus, we can only rely on previous studies (Xing and Isaacowitz, 2006; van Reekum et al., 2007) to infer that age-related differences in emotion regulation goals account for the observed age group differences in fixation patterns, as we did not directly assess or manipulate emotion regulatory goals.

Third, we did not directly assess potential cognitive impairment in the older adult group; thus, we cannot examine the influence of this factor. We refrained from including such a measure to keep the experimental session as short as possible. Given that this group of older participants consisted mainly of students of the guest auditor program for elder persons at the university or participants of the adult education center, we did not expect cognitive impairments. However, even if (mild) cognitive impairments were present in the sample, the testing of our hypotheses would be more conservative because – as we argue – cognitive resources would be necessary for the emergence of the positivity effect (in the present study at a low arousal level). Still, the inclusion of a measure of, for instance, executive functioning in future studies would be interesting to investigate possible interaction effects with the arousal level of stimuli.

Finally, the percentage of female participants is relatively high in the present study. Although Reed and Carstensen (2012) and Reed et al. (2014) do not report gender as a moderator of the PE, future studies should increase the number of male participants.

## Conclusion

The results of the present study emphasize the role of stimulus-related arousal as a moderator of the PE in attention. Older adults paid less attention to low-arousing negative pictures than young adults but this difference reversed for high-arousing stimuli. While age group differences in attention allocation were unrelated to age group differences in immediate emotional responses in terms of pleasantness ratings, they were related to age differences in general negative affect. These findings lend support for the link between age-related changes in attentional processes during affective information processing (as expressed in the PE) and age-related stability of or even increase in emotional well-being.

## Author Contributions

CK, BS, and KF-S: Contributed in conception and design of the work. BS and KF-S: Contributed in writing code and implementing parts of the study in MatLAB. CK: Contributed in writing code and implementing parts of the study in e-prime and Unipark. KD: Collected the data. CK, BS, KD, and KF-S: Contributed in data analysis and interpretation of data. CK and BS: Contributed in drafting and revising work. KF-S and KD: Revised the work.

## Conflict of Interest Statement

The authors declare that the research was conducted in the absence of any commercial or financial relationships that could be construed as a potential conflict of interest.
