# Supplementary material for: Linking the Positivity Effect in Attention with Affective Outcomes: Age Group Differences and the Role of Arousal
Source: Front Psychol. 2017 Oct 30;8:1877. doi: 10.3389/fpsyg.2017.01877 (PMC5670155; doi:10.3389/fpsyg.2017.01877)
Supplement: Supplementary file 1 [file Appendix_B.DOCX]

**Appendix B**

Number of IAPS pictures used in the present study, identified AOI per picture, frequency of chosen AOI and valence ratings for this AOI.

| **IAPS** | **Category Young Adults** | **Category Old Adults** | **AOI-number** | **AOI Choice**  **(%)** | **Valence** | |
| --- | --- | --- | --- | --- | --- | --- |
|  |  |  |  |  | ***M*** | ***SD*** |
| 1120 | NHA |  | 16 | 34.80 | 2.70 | 1.57 |
| 1274 | NHA |  | 26 | 12.87 | 2.00 | 1.18 |
| 1670 | neutral |  | 7 | 44.80 | 5.76 | 1.52 |
| 1811 | PLA |  | 28 | 24.83 | 5.85 | 2.09 |
| 1930 | NHA |  | 14 | 32.15 | 2.06 | 1.48 |
| 1999 | neutral |  | 23 | 28.27 | 5.58 | 1.79 |
| 2141 | NLA |  | 32 | 47.33 | 1.90 | 1.15 |
| 2208 | PLA | PHA | 32 | 27.19 | 7.24 | 1.09 |
| 2278 |  | NHA | 22 | 47.06 | 3.20 | 1.76 |
| 2377 | neutral |  | 12 | 22.32 | 5.16 | 1.68 |
| 2381 |  | neutral | 16 | 44.91 | 5.36 | 1.14 |
| 2383 |  | neutral | 22 | 40.02 | 4.70 | .71 |
| 2393 |  | neutral | 6 | 26.86 | 5.36 | 1.14 |
| 2398 |  | PLA | 13 | 37.03 | 6.39 | 1.55 |
| 2480 |  | neutral | 17 | 44.69 | 5.12 | .53 |
| 2490 |  | NLA | 11 | 30.75 | 3.55 | 1.65 |
| 2495 | neutral |  | 17 | 51.97 | 4.66 | 1.77 |
| 2530 |  | PLA | 16 | 55.54 | 7.80 | .96 |
| 2590 | NLA |  | 7 | 31.54 | 4.57 | 1.63 |
| 2595 | neutral |  | 27 | 25.83 | 4.63 | 1.44 |
| 2616 | PLA |  | 26 | 53.33 | 2.96 | 1.27 |
| 2682 | NHA |  | 14 | 35.28 | 2.57 | 1.50 |
| 2691 | NHA |  | 17 | 39.72 | 5.68 | 1.77 |
| 2720 | NLA |  | 18 | 27.94 | 2.46 | 1.57 |
| 2722 |  | NLA | 16 | 29.66 | 2.34 | 1.41 |
| 2745 |  | neutral | 16 | 14.71 | 4.59 | .71 |
| 2749 |  | neutral | 18 | 12.19 | 2.92 | 1.08 |
| 2753 | NHA |  | 12 | 27.87 | 2.53 | 1.25 |
| 2850 | neutral |  | 17 | 50.66 | 3.88 | .99 |
| 2880 | neutral |  | 27 | 33.18 | 5.21 | 1.07 |
| 3220 | NLA | NHA | 18 | 25.92 | 5.05 | 1.19 |
| 4626 |  | PLA | 22 | 42.91 | 8.05 | .99 |
| 4659 |  | PHA | 8 | 22.18 | 1.91 | 1.51 |
| 4668 | PHA | PHA | 3 | 44.80 | 7.47 | 1.49 |
| 4670 | PHA | PHA | 18 | 21.86 | 6.87 | 2.33 |
| 4680 | PHA | PHA | 18 | 43.68 | 7.22 | 1.61 |
| 4687 | PHA |  | 23 | 36.27 | 7.24 | 1.61 |
| 5130 | NLA | NHA | 18 | 50.51 | 3.61 | 1.43 |
| 5470 |  | PLA | 27 | 25.90 | 6.77 | 1.90 |
| 5531 | neutral |  | 17 | 25.52 | 4.22 | 1.65 |
| 5533 | neutral | neutral | 17 | 19.10 | 6.85 | 1.35 |
| 5534 | neutral |  | 20 | 12.56 | 4.25 | .97 |
| 5535 |  | neutral | 12 | 36.91 | 6.00 | 1.24 |
| 5621 | PHA |  | 17 | 41.02 | 6.32 | 1.89 |
| 5623 | PLA |  | 11 | 35.37 | 6.33 | 1.51 |
| 5626 | PHA | PHA | 14 | 15.37 | 6.75 | 1.44 |
| 5628 | PLA | PLA | 27 | 62.67 | 5.65 | 2.01 |
| 5700 |  | PLA | 11 | 24.83 | 6.57 | 1.53 |
| 5994 |  | NHA | 11 | 20.06 | 7.10 | 1.44 |
| 7000 | neutral |  | 9 | 10.49 | 5.17 | 1.03 |
| 7001 |  | neutral | 22 | 20.49 | 7.23 | 2.05 |
| 7004 |  | neutral | 24 | 37.03 | 5.54 | 1.14 |
| 7010 | neutral | neutral | 8 | 22.00 | 4.95 | .80 |
| 7020 | neutral | neutral | 18 | 44.71 | 5.07 | 1.27 |
| 7023 |  | NLA | 12 | 18.21 | 5.12 | .54 |
| 7025 | neutral | neutral | 17 | 38.10 | 5.12 | .93 |
| 7035 |  | neutral | 22 | 17.24 | 3.25 | 1.44 |
| 7038 | neutral |  | 29 | 10.01 | 2.90 | 1.60 |
| 7039 | neutral |  | 30 | 15.33 | 4.84 | .96 |
| 7040 | neutral |  | 18 | 16.78 | 4.39 | 1.09 |
| 7041 |  | neutral | 17 | 17.43 | 5.69 | 1.35 |
| 7050 | neutral |  | 17 | 30.75 | 4.71 | 1.35 |
| 7055 |  | neutral | 3 | 22.67 | 4.94 | .24 |
| 7057 | neutral |  | 13 | 25.13 | 6.21 | 1.41 |
| 7090 |  | neutral | 14 | 33.92 | 5.00 | .67 |
| 7095 | neutral |  | 21 | 10.73 | 5.36 | .50 |
| 7100 |  | neutral | 19 | 16.45 | 6.19 | 1.35 |
| 7140 | neutral | neutral | 3 | 32.85 | 5.31 | 1.08 |
| 7150 |  | neutral | 23 | 19.68 | 4.92 | 1.19 |
| 7161 | neutral | neutral | 29 | 16.25 | 4.64 | .81 |
| 7170 | neutral |  | 17 | 41.33 | 4.58 | 1.30 |
| 7180 | neutral |  | 17 | 18.10 | 5.79 | 1.40 |
| 7182 |  | neutral | 18 | 46.32 | 5.41 | 1.42 |
| 7184 |  | NHA | 22 | 44.01 | 4.61 | 1.38 |
| 7186 |  | neutral | 18 | 32.12 | 4.43 | 1.52 |
| 7211 |  | neutral | 12 | 17.44 | 4.88 | .62 |
| 7217 |  | neutral | 18 | 15.23 | 5.25 | .80 |
| 7235 |  | neutral | 18 | 25.50 | 4.96 | .45 |
| 7236 | neutral | neutral | 18 | 56.00 | 5.00 | 1.35 |
| 7286 | neutral |  | 24 | 21.99 | 6.29 | 1.52 |
| 7320 | neutral |  | 23 | 17.82 | 5.47 | 1.45 |
| 7359 | NLA |  | 33 | 32.64 | 1.76 | 1.39 |
| 7390 | neutral |  | 7 | 30.52 | 5.63 | 1.38 |
| 7410 |  | neutral | 18 | 17.18 | 6.19 | 1.22 |
| 7430 | neutral |  | 17 | 21.11 | 5.97 | 1.94 |
| 7470 | neutral |  | 14 | 15.75 | 6.69 | 1.89 |
| 7493 | neutral |  | 23 | 26.34 | 7.06 | 1.92 |
| 7499 | PHA |  | 25 | 9.09 | 7.00 | 2.00 |
| 7504 | neutral |  | 24 | 21.42 | 4.87 | 1.29 |
| 7550 |  | neutral | 7 | 29.01 | 4.70 | .84 |
| 7620 |  | PLA | 18 | 18.64 | 4.40 | 1.39 |
| 7660 | PHA |  | 16 | 16.44 | 6.58 | 1.22 |
| 7705 |  | neutral | 23 | 21.92 | 6.00 | 1.54 |
| 7920 |  | NHA | 18 | 17.52 | 3.00 | 1.05 |
| 7950 |  | neutral | 16 | 21.69 | 4.74 | .69 |
| 8030 |  | PHA | 24 | 17.00 | 6.13 | 1.26 |
| 8040 |  | PHA | 12 | 21.43 | 4.55 | 1.60 |
| 8130 |  | PLA | 22 | 44.71 | 5.51 | 1.39 |
| 8185 | PLA |  | 19 | 21.96 | 6.54 | 1.38 |
| 8200 | PLA |  | 21 | 29.58 | 6.46 | 1.33 |
| 8210 | PLA |  | 9 | 40.92 | 6.40 | 1.61 |
| 8510 |  | neutral | 8 | 13.36 | 5.67 | 1.67 |
| 9000 |  | NLA | 20 | 23.00 | 2.86 | 1.75 |
| 9041 |  | NHA | 12 | 31.78 | 3.39 | 2.39 |
| 9090 |  | NLA | 28 | 32.92 | 3.30 | 1.51 |
| 9190 | NLA |  | 18 | 38.06 | 3.30 | 1.68 |
| 9220 |  | NLA | 16 | 28.92 | 2.79 | 1.62 |
| 9280 |  | NHA | 22 | 21.69 | 2.32 | 1.03 |
| 9331 |  | NLA | 11 | 22.78 | 3.80 | 1.36 |
| 9390 | NLA |  | 14 | 27.66 | 3.15 | 1.49 |
| 9395 |  | NLA | 13 | 18.75 | 3.00 | 1.64 |
| 9621 | NHA |  | 13 | 11.19 | 3.58 | 1.56 |
| 9700 | NHA |  | 17 | 24.09 | 5.14 | 1.35 |

*Note*. For each AOI, the table shows the percentage of participants who chose this AOI in a separate rating study and their mean valence ratings for this AOI. NLA = negative low-arousing, NHA = negative high-arousing, PLA = positive low-arousing, PHA = positive high-arousing.
