# Supplementary figures and images for: Linking the Positivity Effect in Attention with Affective Outcomes: Age Group Differences and the Role of Arousal
Source: Front Psychol. 2017 Oct 30;8:1877. doi: 10.3389/fpsyg.2017.01877 (PMC5670155; doi:10.3389/fpsyg.2017.01877)

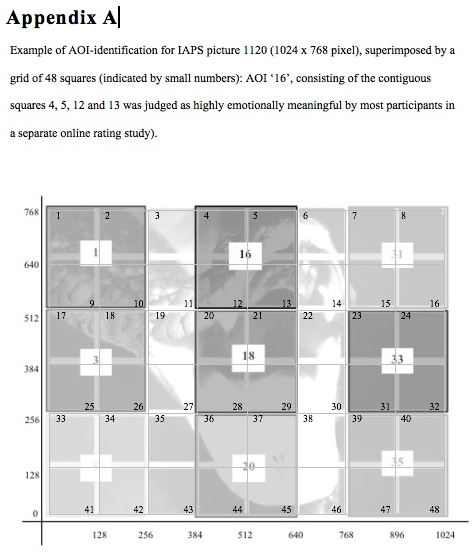

Supplement: Supplementary file 2 [file Appendix_A.jpg]
